# SSRIs: exploring controversies, conundrums, and contentions—epiphenomenon or empirical ingenuity? A comparative panorama for untangling enigma in children and youths

**DOI:** 10.1017/S1092852925000161

**Published:** 2025-03-10

**Authors:** Mayank Gupta, Theodore A. Petti

**Affiliations:** 1 Southwood Children Behavioral Health, Pittsburgh, PA, USA; 2 Rutgers-Robert Wood Johnson Medical School, Piscataway, NJ, USA

**Keywords:** Selective serotonin reuptake inhibitors, psychiatric disorders, monoamine theory, youth and adolescents, mental health, controversies, box warnings

## Abstract

The role of serotonin reuptake inhibitors (SSRIs) in treating psychiatric disorders has been a subject of heated debate since their introduction. Initially celebrated for their potential to address various mental health conditions during the late 1980s and 1990s, SSRIs have since faced significant scrutiny. Critics argue that their benefits may not be as substantial as initially believed. Over the past two decades, concerns have intensified with the emergence of boxed warnings about the risks associated with SSRIs, particularly regarding their link to increased suicidal thoughts in youth. This controversy is further complicated by questions about the integrity of early industry-sponsored trials and the reliability of subsequent National Institute of Mental Health (NIMH) trials. These issues have raised ongoing critical concerns about the effectiveness and safety of SSRIs, especially for treating a range of disorders in children and adolescents. This review seeks to critically appraise by presenting empirical evidence that addresses these controversies. It explores the validity of the monoamine theory, examines the fidelity of early and recent trials, and considers the broader implications for clinical practice. By answering specific, targeted questions, this article aims to clarify the ongoing debate and enhance the understanding of SSRIs’ role in mental health treatment. The goal is to support clinicians in making more informed decisions when prescribing these medications and to ensure consideration of the balance between potential benefits and risks for young patients with mental health disorders.

## Introduction

Freud’s quote, “From error to error, one discovers the entire truth,” resonates in the context of comprehending heterogeneous mental disorders. Psychiatric illness, especially in the pediatric population, is characterized by polygenic inheritance, epigenetic pathways, and heterotopic continuity. Inquiries into the ontology of mental disorders trace their roots back through centuries of human existence. Amidst the critiques directed at the twentieth-century psychoanalytical movement, a significant paradigm shift occurred with the introduction of the Diagnostic and Statistical Manual-III (DSM-III). This shift aimed to depart from theoretical underpinnings, embrace a theoretical categorical nosology for mental disorders, and facilitate a foundation for empirically driven research. However, the 1970s overhaul of DSM-III, while establishing a categorical nosology, revealed subsequent gaps in the contextual comprehension of psychiatric illnesses. Criticisms rooted in pure reason have endured for the past two centuries. Initial steps in developing research hypotheses necessitate *Socratic Questioning* to formulate precise inquiries addressing biases, confounders, the interpretation of statistical signals, and the limitations of study designs.

Serotonin (5-hydroxytryptamine [5-HT]) was initially isolated about 80 years ago and later discovered in the brain in the early 1950s. This led to the speculation in the late 1950s that abnormal serotoninergic functioning might be implicated in certain mental disorders Top of Form concurrent with the introduction of the serotonin hypothesis.^1^ The evolutionary timeline of antidepressants extends back to the 1960s, marked by the advent of monoamine oxidase (MAO) inhibitors and tricyclic antidepressants (TCA).

Selective serotonin reuptake inhibitors (SSRIs) received United States Federal Drug Agency (FDA) approval in 1987, entering the market in January 1988. Subsequently, throughout the 1990s, their indications increased to encompass a spectrum of treatment for affective illness, trauma, and anxiety disorders and expanded into the realm of more medically related illnesses.^2^ ^,^^3^

However, the initial promotional enthusiasm lauding the sanctity of SSRIs was short-lived. Discernible statistical signals prompted multifaceted inquiries about safety and elicited adverse media coverage and public health deliberations.^4^ ^,^^5^ During the 1990s, industry-sponsored placebo-controlled trials were pivotal in securing FDA approvals. Simultaneously, controversies emerged from Study 329, a clinical trial conducted in the US from 1994 to 1998, which garnered substantial negative media coverage.^6^ The controversy coincided with a cautionary alert for paroxetine issued by the Medicines and Healthcare Products Regulatory Agency in the United Kingdom in May 2003.^7^ While the FDA initially issued a cautionary advisory based on related findings, a subsequent meta-analysis examining 24 SSRIs and other newer-generation antidepressants for suicidal events from placebo-controlled pediatric trials,^8^ led to the “black box” FDA warning for all SSRIs by October 2004.

This was followed by counterarguments suggesting FDA over-reach, overreaction, and then the emergence of increases in adolescent deaths by suicides posited as probably secondary to decreases in SSRI prescribing following the warning.^7^ Moreover, a significant threat to accurate risk-assessment analyses from inconsistent labeling of potentially suicidal events was identified in the analyzed data. A panel of experts from Columbia University conducted a study to scrutinize these assertions. The Columbia Classification Algorithm for Suicide Assessment (C-CASA) is a standardized suicidal rating system that provides data for the pediatric suicidal risk analysis of antidepressants conducted by the FDA. It concluded that the SSRI risk of suicidality was overestimated^9^ and became a gold standard for assessing suicidality in studies and clinical practice.

**Figure 1.**
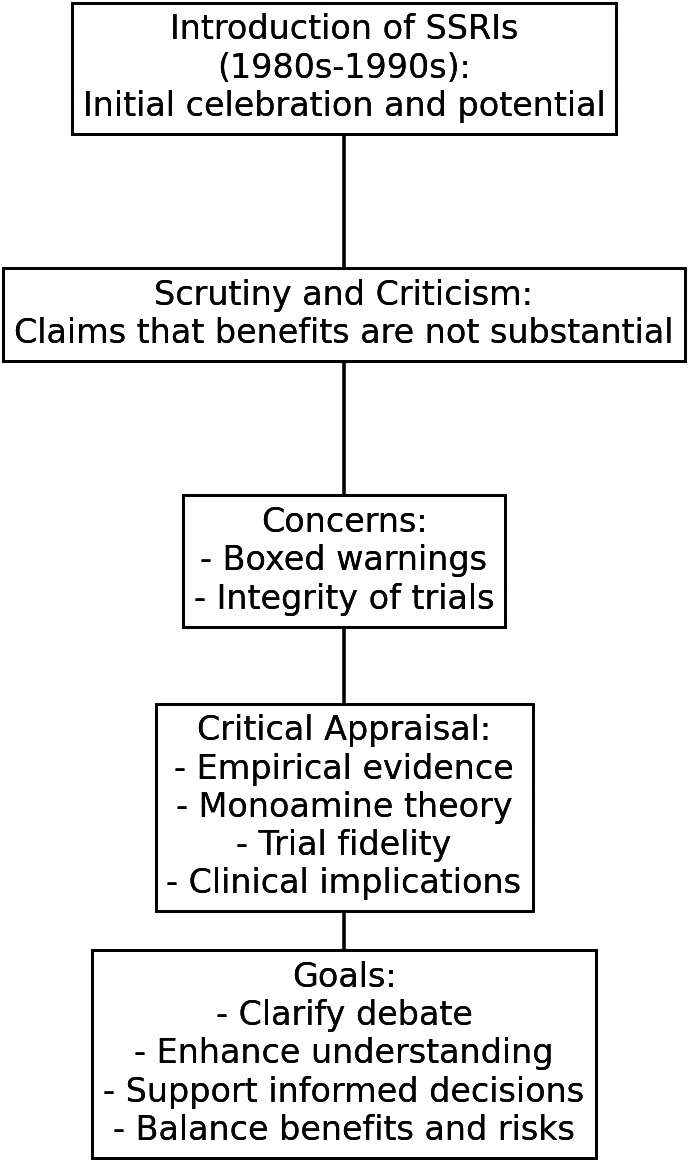
A graphic overview of some critical examinations in review.

Over the years, a myriad of other issues was linked to SSRIs, including the risk of switching to mania, an elevated risk of fractures in the elderly, and the potential adverse effects of antenatal exposure.^10^ However, the relationship between antidepressants and suicidality is not supported by meta-analysis, though monitoring treatment emergent suicidality is recommended. While empirical evidence remains limited, it indicates that combined pharmacotherapy—such as the use of antidepressants and/or lithium—alongside psychotherapy may be effective in reducing pretreatment suicidal ideation and mitigating suicidal risk.^11^ These controversies were subjected to rigorous scientific dialectical processes, reflecting the multifaceted dimensions of the discourse surrounding SSRI medications. The meta-analyses comparing SSRIs to placebo have led to inquiries concerning both the lack of efficacy and the risk of behavioral activation, and posing the potential for harm in individuals with autism spectrum disorder (ASD) and other disorders.^12^ These schisms in perspectives were debated among researchers, clinicians, advocates, and the public. The figure 1 presents a temporal analysis of SSRI-related controversies, tracing the chronological emergence of evidence and identifying critical areas for further research and clinical advancements.

Therefore, many critical questions must be addressed. First, do SSRIs exhibit clinically significant efficacy in the pediatric population? If yes, what are the specific clinical conditions for which SSRIs demonstrate effectiveness? Is efficacy localized to specific SSRIs, and to what extent are criticisms by skeptics valid? Second, what constitutes the robust empirical evidence substantiating the efficacy of SSRIs, and what are its recognized limitations? Those limitations include adverse effects, an area too infrequently considered,^13^ eg, the concept of behavioral activation: is there a neuroscientific underpinning of behavioral activation, and is there a discernible demographic group that is particularly impacted by its effects? These and related questions demand attention for safe and effective SSRI use.^12^

Owing to the diverse array of evolving variables and confounding factors, numerous critical distinctions exist in the therapeutic applications of SSRIs for adults, children, and adolescents. The developmental dimension of psychiatric phenomenology, the validity of diagnostics, the inadequate psychometrics of scales for screening and diagnoses for which SSRIs can be used,^14^ the temporal aspects related to the delay or attainment of developmental stages, the differentiation between normative and psychopathological symptoms, the ontogeny of receptors, and the intricate pharmacokinetic and pharmacodynamic profiles represent only a subset of the multifaceted and challenging variables involved in this context, which will be addressed. Table 1 provides a comprehensive overview of several critical debates regarding the use of SSRIs in children and adolescents, highlighting key concerns, benefits, and ongoing controversies in clinical practice and research.

**Table 1.** Scientific responses to SSRI-related concerns and some

Critical debates	Controversies	Scientific disagreements and variability in interpretations
1. Onset of SSRI efficacy	The downregulation of 5HT1A auto-receptors typically takes 3–4 weeks. However, the exact mechanisms remain unclear.	Meta-analysis suggests a possible response as early as 1 week, with some drugs like Mirtazapine showing faster action due to atypical effects on 5HT2A receptors.
2. Understanding major depressive disorder (MDD)	The Monoamine Hypothesis lacks conclusive evidence, and MDD exhibits diverse phenotypic and genetic pathways.	Methodological limitations in studying mental disorders hinder causal understanding, necessitating a broader perspective.
3. Clinical phenotypes in children and adolescents	Developmental differences in symptom presentation and receptor maturation underscore the need for nuanced assessment.	Overlapping symptoms necessitate a careful assessment of symptom onset chronology to guide treatment strategies.
4. Long-term efficacy and safety	Industry trials have shorter durations, limiting long-term data availability.	Statistical separation from placebo is observed within 2–4 weeks, but challenges in outcome measurement and reliability of diagnoses persist.
5. Placebo vs. head-to-head trials	Methodological differences influence placebo responses, with head-to-head trials showing fewer placebo effects.	Strategies such as active placebo use and robust inclusion criteria mitigate placebo effects, providing clearer drug differentiations.
6. Unique mechanisms of G protein-coupled receptors (GPCRs) in SSRI action	Short-term and long-term desensitization mechanisms influence SSRI action on GPCRs.	Desensitization processes provide insights into the delayed onset of SSRI effects.
7. Placebo response in comparison to other conditions	SSRIs demonstrate superior efficacy compared to many medications for physical health issues.	Harnessing placebo responses underscores the importance of monitoring treatment response over time for effective management.

This two-part paper considers various critical SSRI-related issues to develop a systematic dialectical approach that utilizes a priori deductive and inferential processes. Given the material’s density, the first paper attends to theoretical and empirical gaps while the second paper provides systematic approaches to utilize in clinical settings. We strive to offer clinicians an informative review of the literature, to assist them in making shared medical decisions with their patients when considering SSRI interventions.

## Methods

This narrative review explores the controversies surrounding SSRI use in children and adolescents. Unlike systematic reviews, it does not follow MOOSE guidelines or have a prospective registration with PROSPERO. Instead, a comprehensive search was conducted across various databases, including PubMed, PsycINFO, the Cochrane Library, Google, and Google Scholar, using reverse citation searches through January 2024. Keywords such as “SSRI,” “depression,” “anxiety,” “serotonin,” “selective serotonin reuptake inhibitors,” “FDA,” “antidepressants,” and “controversies” were employed, with filters applied to focus on child and adolescent populations. Only articles published in English, regardless of geographic location, were considered.

The inclusion criteria targeted studies addressing critical topics like the monoamine hypothesis, boxed warnings, effect size, and placebo response in the context of SSRI use. To ensure a comprehensive review, manual searches, including those on PubMed Central, were conducted. Emphasis was placed on references that summarized earlier research, such as reviews and meta-analyses, with individual studies cited when necessary to support or elaborate on key points. This approach was designed to provide a broad perspective on the ongoing debates regarding SSRI use in younger populations. Initially, 17 articles were identified, with an additional 62 articles added through manual searches and reverse citation tracking, resulting in a total of 79 articles included in this review.

## Results and discussion

### Serotonin, serotonin receptors, serotonin transporters, and the puzzle: why consider SSRIs?

Serotonin (5-HT) plays a crucial role in early life to regulate various developmental processes associated with neural circuit formation. Animal studies involving rodents exposed to SSRIs during brain development have shown that dysregulation of early-life 5HT leads to a broad spectrum of psychiatric-relevant phenotypes.^15^ Given the need to appraise these complex processes, a brief diversion to basic neurodevelopment principles is warranted.

In addition to genetic alterations, epigenetic processes like early-life stress can impact the migration of cortical interneurons.^16^ The identification of its receptor subtypes, the serotonin 1A receptor (5HT1AR), serotonin 2A receptor (5HT2AR), and the serotonin transporter (SERT) contributed to a more nuanced understanding of myriad parallel pathways to the GABAergic system. These pathways engage various brain circuitries, each playing a distinct role in regulating human emotions and behaviors. Specifically, 5HT1AR influences subcortical responses to stress, while 5HT2AR plays a crucial role in modulating the prefrontal cortex (PFC). While 14 different 5HT receptors are identified, it is the role of 5HT1AR downregulation in the somatodendritic areas of the serotonergic neurons that is considered the primary target for SSRIs.^17^ SERT blocking the reuptake of 5HT happens shortly after initiation of SSRIs, however, the process of the 5HT disinhibition and subsequent firing of hippocampus postsynaptic 5HT1A receptors are the core targets^19^ that can include both inhibitory and excitatory functions. Many studies have debunked the myth that it takes 3–4 weeks for SSRI’s effect to be observed, as two meta-analyses have demonstrated effects as early as a week.^18^ ^,^^19^

The promoter region of 5HTR2AR undergoes epigenetic modification through methylation during fetal development. These modifications have been linked to neurobehavioral problems in infancy, psychiatric disorders in young adulthood, and chronic fatigue syndrome in adults. Stress has an integral role in the epigenetic regulation of HTR2A, given that both glucocorticoid and cortisol binding sites are present in the promoter region.^20^ The serotonin 2C receptor (5HTR2C) is also a target for most antidepressants and atypical antipsychotic medications. Polymorphisms of the 5HTR2C gene have been linked to an elevated risk of experiencing side effects such as weight gain and movement disorders associated with the use of atypical antipsychotics^21^ and weight gain or loss with specific SSRIs.^22^

The serotonin transporter (SERT) that is responsible for serotonin reuptake into the presynaptic neuron is encoded by the SLC6A4 gene at Ch17q11.1-q12 and is one of the most extensively studied pharmacodynamic genes. It possesses both short and long polymorphisms of the gene promoter allele.^20^ ^,^^23^ The SERT plays a crucial role in shuttling through the synaptic cleft from the serotonergic neuron to the post-synaptic receptor, collecting and recycling unbound serotonin. The *homozygous form of the short allele (S/S)* is linked to reduced transcription efficiency, lower gene expression, and decreased serotonin binding affinity.^24^ Both human and animal studies have demonstrated that individuals with the *S/S genotype* exhibit increased sensitivity to stress from adverse life events. The *SS genotype* is associated with anxious, neurotic, and somatic personality styles and with serious, more frequent side effects.^25^ The homozygous form of the *long allele (L/L)* is linked to higher gene expression and resilience. The empirical data supports the argument that SSRIs bind with variable affinity to the SERT, displacing serotonin into the synaptic cleft; the *SS genotype* is associated with decreased response to SSRI medications, while the *LL genotype* is linked with increased response.^26^

The postsynaptic 5-HT1AR is densely organized in limbic regions, particularly the hippocampus. The hippocampus has a dense expression of 5HT1A receptors on excitatory neurons. Stimulation of 5-HT1AR has an inhibitory effect on pyramidal neuron activity, and serotonin appears to mitigate limbic (hippocampus) hyperactivity through the inhibitory action of postsynaptic 5-HT1ARs.^27^ The seminal role of 5HT2A receptors has fostered opportunities for novel preventive and interventive approaches, given the signaling of 5-HT2AR during highly sensitive periods of plasticity that are environment-dependent.^28^ This hypothesis gains support from the observation that the expression of central 5-HT2ARs is most pronounced during critical developmental periods coinciding with the peak of plasticity-related learning.^28^ We consider SSRIs due to their impact on emotional and other affective states during critical developmental periods.

### Assessing the efficacy of SSRIs (do they work?)

The heterogeneity that anxiety and depression express as related to etiology, presentation, and efforts at measuring and preventing or treating to reduce their severity may be why SSRIs were considered and tested in clinical practice. Questions posed with “How” aim to employ an empirically driven approach to answer and substantiate hypotheses regarding the mechanisms of SSRI functioning. Utilizing models involving 5-hydroxyindole acetic acid (5-HIAA), SERT, and 5HT1AR, numerous questions persist, eg, medical models including the elusive understanding of how acetaminophen operates. These critical issues are addressed in the next section.

On the other hand, questions framed with “Does” offer a more focused inquiry that, when subjected to a robust and methodologically sound model, hold the potential to yield informative outcomes to clinical care. The inherent challenge in studying human “subjectivity” as a unitary construct lies in its limitations, influenced by a myriad of biases and the need for tailored methodologies with challenges to generalize for individualized care. Nevertheless, each question, when systematically investigated, contributes factual insights to guide the application of inductive and deductive reasoning in the realm of medical decision-making for complex clinical and developmental phenotypes.

Among many barriers, critiques of a strong placebo effect in SSRI trials are a matter of serious contention among stakeholders. Mean placebo response rates in early antidepressant clinical trials range from 30% to 40%, surpassing those observed in other mental disorders.^29^ Attributed partially to the quality and methodology of studies. This has led to subsequent systematic changes in study designs to address methodological problems. The inclusion criterion of numerous industry-sponsored studies conducted from the 1990s to 2010 in recent analyses is questionable, given their persistent, elevated placebo signals contributing to the conundrums.^30^ ^,^^31^

These controversies persisted until 2010, attributable to a dearth of newer SSRI studies that controlled placebo responses in the meta-analysis.^32^ Outcome measures in those studies continued to focus on symptom reductions and functionality. This underscores the limitations of attempts to objectively measure psychiatric symptoms while accounting for the subjective aspects. This challenge arises due to the complex, heterogeneous, multifactorial, and pleiotropic etiology of mental disorders. Inherent flaws in statistical design are posited to have impeded the capture of measurable differences in placebo-controlled trials. Though the high placebo response is considered elsewhere in this paper, as likely due to the higher number of study sites and less reliable enrollment procedures in industry versus government or academic-supported studies, the heterogeneity of depressive etiologies also contributes to significant, alternative explanations.^11^

A study involving 116,477 adult patients examined and found that all 21 antidepressants were significantly more effective than placebos in reducing the severity of depressive symptoms. This was defined as at least 50% from baseline in adults with major depressive disorder (MDD) after around 8 weeks.^33^ The odds ratios ranged from 1.37 to 2.13, favoring antidepressant use. Some critics argue that the effects highlighted in meta-analyses may lack clinical significance and suggest that side effects could potentially confound the results.^34^ Nevertheless, examinations of subjective mood have indicated distinct advantages for certain antidepressants over placebos, even in situations where side effects are absent.^35^

A broad comparison against placebos demonstrated antidepressants having greater effectiveness than numerous medications addressing physical health issues. For instance, the number needed to treat (NNT) for antidepressants and depression is typically 3 or 4, which is lower than the NNT for routinely prescribed medications for other medical illnesses, eg, ACE inhibitors for hypertension and chronic heart failure, thrombolytic drugs for acute stroke, and metformin for diabetes.^36^ ^,^^37^ The NNT of 4 for major depression comes from the Treatment for Adolescents and Depression Study (TADS), the major NIMH-sponsored adolescent depression trial that showed that fluoxetine was more highly effective over the 12-week acute phase. Moreover, over 9 months ~80% of adolescents improved.^38^

This must be contrasted to the equally impressive NNT of 10 for positive response with depressed teens reported in the earlier meta-analysis conducted by Bridge and associates.^39^ It included large placebo response rates (~60%) from the many industry-sponsored trials for MDD in pediatric patients, resulting in estimates of an NNT closer to 20 from smaller between-group differences due to large placebo responders. The differences relate to methodological rigor.^30^ The expectancy of a positive response to explain differences between response rates between pediatric and adult patients appears less important in medication treatment for depressed youth as it has been demonstrated for depressed adults.^40^

Therefore, the question is answered affirmatively—SSRIs work when appropriately assessed. The caveat is that methodology counts heavily in pediatric psychopharmacology studies! The next set of questions then is how does this happen and why.

### SSRIs and the monoamine hypothesis—How do they apply?

During the 1960s, the monoamine hypothesis was formulated by J.J. Schildkraut and associates. They consider catecholamines, a class of naturally occurring compounds that act as messenger chemicals within the brain.^41^ The serotonin theory of depression was based on a chemical imbalance paradigm but lacks empirical substantiation as critics question the widespread adoption of this theory in the public domain.^42^ Experts argue that it was explicitly presented as a heuristic hypothesis rather than a fully developed theory. Its later clarification as a correlational hypothesis, characterized it as an association rather than implying causation, became the focus of scathing scientific critiques.^43^ ^,^^44^

Between 1990 and 2010, a minimum of 17 alternative hypotheses related to depression emerged, and an additional 8 have been proposed since.^44^ These theoretical models encompass neurophysiological, neuroendocrine, chronobiological, immunological, morphological, and other perspectives. While the monoaminergic hypothesis, involving serotonin and/or norepinephrine, remained a subject of continuous discussion, debate, and modifications among researchers during this period, it never again gained widespread acceptance as a causal theory for mood disorders.^44^

The debate extends to ambiguity about the role of tryptophan and 5HIAA. Two meta-analyses focused on circulating tryptophan concentrations, which directly impact central 5HT synthesis.^45^ ^–^^49^ Tryptophan is considered more pertinent to the central nervous system’s 5HT function compared to 5HT and 5-HIAA levels in body fluids, ie, L-tryptophan plasma concentrations exhibit a significant decrease in individuals with MDD after adjusting for publication bias (Hedge’s g = −0.45, 95% CIs, −0.66 to −0.23). Among unmedicated individuals, the effect size is notably large, with g = −0.84 (95% CIs −1.27 to −0.4).^45^

The 5-HT1AR signaling also plays a crucial role in learning and cognition. Postsynaptic 5-HT1AR stimulation is believed to be the key therapeutic site of action for SSRIs. Prolonged use of SSRIs has been linked to increased neurogenesis, particularly in the hippocampus, with observed enhancements in learning and cognition.^32^ ^–^^34^ ^,^^50^ However, exceptions have been documented. Several case reports and studies document cognitive-related problems, particularly memory-related.^51^ ^,^^52^ Animal models demonstrate similar SSRI positive and negative impacts on cognitive functioning with issues related to specific compounds.^53^ ^,^^54^ This may relate to the effect on the hippocampus relating to deprivation of total and rapid eye movement (REM) sleep playing a significant role in modulating passive avoidance memory.^55^ Sleep deprivation-induced memory impairments can result from up or down-regulation of serotonergic receptors during training for memory tasks.^56^

Though 5HT1ARs are assumed to operate exclusively as presynaptic autoreceptors, most 5-HT1ARs are postsynaptic heteroreceptors.^57^ The reduced availability of post-synaptic 5-HT1ARs in unmedicated depression would align with the concept of lowered 5-HT neurotransmission. In one study, middle-aged healthy volunteers were provided with a tryptophan-rich diet for 19 days.^58^

Results indicated a reduction in emotional bias towards negative stimuli among the participants. Furthermore, evidence suggests that the neuroplasticity effects of SSRIs become apparent only following a more prolonged administration period.^59^ Short-term usage of antidepressants has shown potential in alleviating the negative biases in information processing commonly associated with mood and anxiety disorders.^60^

The Therapeutic effects of interventions seem to be influenced by subsequent neurophysiological changes. These changes include the differential expression of monoaminergic receptor levels, downstream intracellular effects on metabotropic enzyme cascades, and subsequent alterations to the nuclear transcription of proteins, eg, brain-derived neurotrophic factor (BDNF). Thus, the impact of therapeutic interventions on the brain’s neurochemistry and molecular processes plays a crucial role in achieving therapeutic outcomes. The modulation of neurotransmitter receptors and the promotion of factors, eg, BDNF, are part of this process.^61^

MDD and anxiety disorders are frequently linked to a “negative bias” in an individual’s cognitive processing. Cognitive behavioral therapy (CBT) operates, in part, by challenging automatic negative thoughts; interestingly, SSRIs can also alleviate the cognitive bias associated with MDD. Of concern, SRRI treatment might negatively impact through its mediating acute SSRI effects on fear learning by altering glutamatergic transmission. Cued and context fear conditioning would be affected via the amygdala, hippocampus, and bed nucleus of the stria terminalis. The latter is a complex heterogeneous limbic forebrain structure functioning to control autonomic, neuroendocrine, and behavioral responses.^62^

SSRIs’ effect on the functioning of specific brain regions on behavioral and emotional effects has been long studied regarding Pavlovian fear conditioning, a well-characterized model of emotional learning. A proposed model explains why acute SSRI administration may result in initial anxiety by altering neural activity in the extended amygdala and hippocampus. Numerous rodent studies investigating fear learning support the conclusion that SSRIs impair the expression of contextual fear conditioning while allowing the acquisition and expression of cued fear conditioning. Thus, disruptive effects on fear learning should be considered when combining chronic SSRI treatment and CBT and other learning-based therapies to effectively reduce anxiety symptoms.^62^

Likewise, a recent study with 66 healthy volunteers, employing a double-blind placebo-controlled design, reports that emotional blunting is a common side effect of SSRIs. This conclusion is reinforced by a semi-randomized study, balanced for age, sex, and intelligence quotient (IQ) of placebo (*n* = 34), or 20 mg of escitalopram (*n* = 32). The intervention lasting for >21 days found that compared to placebo, escitalopram reduced reinforcement sensitivity on two measures, the Sequential Model-Based/Model-Free task and the Probability and Reversal Learning task. This SSRI thus appears to work by reducing some emotional pain by allowing the individual to become less sensitive to rewards and may explain the often reported “blunting” effect.^63^

The mechanisms by which they exert their effects are complex and involve neurophysiological, neurochemical, neurotransmission, and neuroplastic changes. Alterations in brain function depend on the differential expression of monoaminergic levels, particularly serotonin, and the downstream processes that follow. This could be associated with a reduction in emotional pain.

### Exploring empirical pathways and their challenges in studying heuristic hypotheses

In the annals of healthcare legislation, the year 1997 stands out as a pivotal moment with the introduction of the Food and Drug Administration Modernization Act (FDAMA).^64^ This marked the genesis of a concerted effort to address the gaps in pediatric drug development. The early sections of this narrative revealed the challenges and inadequacies in pediatric drug development; recognizing the need for a more robust approach to pediatric labeling information, legislators made commendable efforts to refine and fortify the regulatory landscape.

The turning point arrived in 2002 with the enactment of the Best Pharmaceuticals for Children Act (BPCA). This legislation emerged as a beacon of change, introducing incentives to encourage pharmaceutical companies to invest in pediatric studies. The narrative gained depth with the introduction of the Pediatric Research Equity Act (PREA) in 2003. This complementary piece of legislation mandated pediatric studies for drugs anticipated for use in children, with the expectation to further enrich the understanding of drug safety and efficacy in pediatric populations.^65^

### Mean group differences and the Hamilton depression rating scale

Until 2004, the flurry of industry-sponsored trials driven by the FDA amendment to include children and adolescents in studies was often criticized for numerous methodological issues, higher placebo responses, and minuscule group differences. This led to many controversies about the true efficacy of SSRIs, with raised scientific doubts often resulting in a media frenzy and denigrating care for millions of individuals benefiting from SSRIs. As noted above, methodological rigor is related to motivation (ie, profit and the need to mainly demonstrate having done the study) and efficacy versus advancing the science, clinical efficacy, and effectiveness.

Kirsch and colleagues, for example, published a comprehensive investigation into the efficacy of antidepressants by analyzing both published and unpublished data from FDA registration trials. Their primary conclusion was that antidepressants did not show clinically significant effects for individuals with mild, moderate, or severe depression.^66^ The mean difference between the drug and placebo groups was only 1.80 points on the Hamilton Rating Scale for Depression (HDRS). Notably, clinical significance was observed only in cases of very severe depression. The suggested attribution for that finding is increased responsiveness to medication in very severely depressed patients and decreased responsiveness to placebo. This is in line with the recommendations to require a level of severity from moderate to severe for treating anxiety disorders, major depression, and obsessive-compulsive disorder (OCD) with medication.^67^

Moreover, the use of the HDRS raises other concerns, particularly regarding its inclusion of items that are not specific to major depression. A typical of this are the six sleep-related items that could be influenced by nonspecific sedative effects associated with many antidepressants.^68^ Consequently, improvement in baseline scores might reflect nonspecific effects rather than actual changes in mood. Additionally, substances, eg, stimulants (methylphenidate), benzodiazepines, and second-generation antipsychotics, have demonstrated antidepressant effects, suggesting that improvement is not exclusive to the unique actions of antidepressants.^69^

In a review of adult studies comparing SSRIs with placebos, 18 out of 32 comparisons did not show significant superiority of the studied SSRIs over placebo with the reduction in HDRS scores. However, only three out of 32 comparisons did not significantly reduce depressed mood (*P* < 0.001).^70^ This suggests that the issue of negative SSRI trials, often cited as a significant point of contention in discussions about the effectiveness of antidepressants, may be partly attributed to employing inadequate sensitive measures to assess trial efficacy results and their interpretation.^14^

Relying solely or predominantly on these analyses or industry-sponsored studies may result in an underestimation of antidepressant efficacy. This emphasizes the importance of using a comprehensive approach that goes beyond a single measure for a more nuanced understanding of antidepressant efficacy as well as determining the motivation and implementation of the study.

### Lack of statistical power and inclusion criteria of randomized controlled trials

A Cochrane systematic review notes methodological shortcomings of randomized trials that make findings difficult to interpret SSRI and other newer antidepressant medications’ safety and efficacy. However, it concludes that SSRIs’ impact is relatively insignificant; they do qualify their findings by noting the heterogeneity of depression and concede that greater improvement may be gained by some and that most SSRIs and duloxetine could be considered as first-line treatment.^71^

That shotgun-like approach must be contrasted to the earlier, more thoughtful systematic search by Vitiello and Ordonez, which considers the ongoing debate for depressed youth about the antidepressant cost/benefit balance. They note that 30 related controlled clinical trials are almost a third statistically underpowered and that the only two placebo-controlled and sufficiently statistically powered studies showed fluoxetine efficacy, though one of those demonstrated increased suicidal ideation and behavior (OR = 2.39). They recommend that antidepressants, with their safety concerns and high response to nonspecific interventions in youth, should be used cautiously and limited to those where psychosocial interventions are unavailable or ineffective and limited to moderate-to-severe depression.^67^

Additional concerns have been raised regarding the representativeness of patients in randomized controlled trials (RCTs) compared to the broader depressed population, primarily due to stringent inclusion and exclusion criteria. For instance, individuals with low-severity symptoms, comorbid anxiety, or significant suicidal ideation are often excluded from phase III clinical trials. A study reported that only 22.2% of patients met the eligibility criteria for phase III trials in the Sequenced Treatment Alternatives to Relieve Depression (STAR*D) project. STAR*D deliberately employed broad inclusion criteria to capture a more representative sample of depressed outpatients.^72^ This discrepancy highlights the potential limitations in generalizing findings from clinical trials to the broader population of individuals experiencing depression. The reported outcomes were inflated due to the inclusion of 99 patients who were already in remission based on the Hamilton Rating Scale for Depression (HDRS) at the study’s start, as well as 125 patients who were in remission when beginning the next level of treatment. These patients should have been excluded from the analysis to maintain methodological rigor. The STAR*D study initially reported a 67% cumulative remission rate after up to four antidepressant treatment trials. However, when applying the protocol-defined HDRS criteria and appropriate inclusion parameters, the actual remission rate was found to be 35.0%. This indicates that SSRIs might be more effective in patients with severe depressive symptoms.^73^

### How NIMH studies in children and adolescents debunk earlier conclusions

After serious questions were raised about the high placebo responses with SSRI, low group differences, and effect sizes in the mid-2000s, NIMH-funded studies were conducted while addressing the methodological limitations. The critical fixes included more robust inclusion criteria with a developmental understanding of the group ages, the use of appropriate measures, comorbidity assessments, adequate duration of trials to address initial placebo responses, power calculations, use of active placebo, and concomitantly studying suicidality associated with the SSRI.

By the time the NIMH-sponsored TADS, considered the definitive trial for teen depression, was published in 2004, 14 industry-sponsored trials evaluating newer antidepressants for pediatric depression were already concluded. TADS, the sole extensive direct comparison between fluoxetine and CBT, revealed a significantly higher response rate to fluoxetine (60.6%) compared to psychological therapy (43.2%) at the 12-week mark.^74^

TADS demonstrated the effectiveness of SSRIs, specifically fluoxetine, with an NNT of 4 over the acute phase lasting 12 weeks. Ultimately, around 80% of adolescents showed improvement over the 9-month study period. The TADS study is considered the largest and one of the highest-quality acute-phase randomized placebo-controlled trials of an antidepressant for teen depression. The TADS inclusion criteria required participants to have a stable depressed mood for at least six weeks in at least two out of three settings (home, school, or among peers), minimizing the potential for placebo-responsive mood fluctuations. Also, the primary outcome measures were evaluated by an independent assessor who was blinded to the treatment assignments and course of treatment. This approach might have introduced a bias, potentially attributing improvements to the active treatment. Additionally, unlike other trials that included numerous small clinical sites with limited research experience, all three fluoxetine studies utilized fewer clinical sites.^74^ Therefore, TADS reported a relatively low placebo response rate of 35%. The drug–placebo differences have diminished over time, largely because of methodological changes mixing the groups and adding additional variables.^30^ TADS’ positive effect size and NNT (~4) sharply contrast with industry trials and are more attuned to clinicians’ experiences. These findings underscore the effectiveness of the SSRI intervention in the TADS study for treating teen depression over an extended treatment period.^74^

Notably, many industry-sponsored trials for MDD in pediatric patients reported substantial placebo response rates (~60%), leading to smaller between-group differences and NNT estimates closer to 12. This has contributed to challenges in meta-analyses that encompass all trials. Industry-sponsored trials are often categorized as negative trials, indicating a lack of demonstrated efficacy. However, challenges inherent to the methodology and implementation of these studies suggest that they should be considered failed trials, ie, failure can arise from a lack of efficacy or safety issues related to flawed study design, having an underpowered sample size, inappropriate selection of patient’s illness severity, insufficient funding to complete the trial, and countless other factors.^30^ Therefore, they may be largely uninformative regarding the efficacy and might be deemed ineligible for inclusion in meta-analyses.

There are numerous inquiries regarding the notably high placebo response observed in trials of SSRIs involving children and adolescents. In contrast, studies funded by the NIMH, characterized by methodological strengths, showed lower placebo response rates of 33%–35% and meaningful between-group differences of about 25%^30^ in supporting the efficacy of antidepressants. This highlights the importance of considering the study design and methodology when interpreting trial outcomes and underscores the potential impact of these factors on the perceived effectiveness of antidepressant interventions. Such studies employing sophisticated research methodology, including comparative treatment trials with valid comparison and control groups, were aimed at reducing placebo response rates. The interventions were manualized, and study clinicians underwent rigorous processes of reviewing study materials and demonstrating mastery of the protocol and the intervention under study.

Independent evaluators and incorporating metrics suitable for mediator and moderator analyses significantly improved the studies’ validity. Statistical analyses were conducted using advanced, valid methods, eg, imputation approaches for handling missing data, as opposed to the last observation carried forward.^30^ Throughout the NIMH study implementations, weekly conference calls were common. These calls served various purposes, such as reviewing recruitment strategies and ensuring that the right participants were included in the trial. Groups of principal investigators, study coordinators, evaluators, and clinicians held frequent conference calls to maintain fidelity and quality throughout the research process. Such motivation and commitment to high-quality methodology and implementation are indicative of the careful consideration given to various aspects of the study design, execution, and analysis.^30^

The placebo response in key child and adolescent studies involving SSRIs, including TADS, Child/Adolescent Anxiety Multimodal Study (CAMS), Research Units on Pediatric Psychopharmacology and Psychosocial (RUPP) Anxiety, and RUPP Autism Repetitive Behaviors, was consistently low.^13^ This low placebo response allowed for the identification of meaningful differences between active medication and placebo, ranging from 25% in TADS, 30% in CAMS, to over 50% in RUPP Anxiety. This suggests that the observed improvements in the groups receiving active medication were significant and beyond what could be attributed to the placebo effect alone.

The addition of concomitant CBT in MDD demonstrates improvement in depressive symptoms, although not as robustly as in generalized anxiety disorder (GAD) and OCD.^38^ While the full benefits of SSRIs for depression may take up to 8 weeks to manifest, a meta-analysis of depression studies in pediatric patients suggests that significant placebo benefits can be observed as early as 2 weeks. Furthermore, the analysis indicates that additional treatment gains are minimal after 4 weeks. Therefore, the recommendation is to pursue at least a 4- to 6-week trial at therapeutic dosing before considering a medication as a treatment failure.^38^

If the 5HT system is involved, as its role in many in-vivo studies and neuroimaging studies has been established, determining SSRIs’ effectiveness for highly heterogeneous clinical mental disorders is critical. As the intellectual debate continues, determining their mechanism of action employing a drug-centric rather than a traditional disease-centric approach could answer narrower, critical questions.^75^ The consistent inferences drawn from an RCT for a condition likely to be heterogeneous often relate to whether the treatment is effective for a subgroup. If it is effective for a subgroup, then its generalizability is always in question and could create circular reasoning fallacies.

Study findings from an influential 2006 paper demonstrate psychiatrists’ responsibility for much of outcome variance and conclude that psychiatrists’ approach to treatment can augment the effects of both active anti-depressant medication and placebo. Over 120 patients were divided into two groups: one group received a placebo and another group received full-strength psychiatric medication for depression. At the same time, each psychiatrist was rated on several personal qualities: verbal fluency, interpersonal perception, expressiveness, warmth, acceptance, empathy, and the ability to focus on the other person. When results were analyzed, the psychiatrists strong in these personal qualities had the best results, even when the “medication” they prescribed was a placebo.^76^ Therefore, harnessing the placebo effect is an essential part of clinical practice that warrants attention in data analysis as is attending to the case formulation, the doctor-patient relationship, assuring adequate communication with the patients and their families, and providing the requisite attention and empathy.^77^ ^,^^78^

NIMH funding has significantly and meaningfully advanced our knowledge base addressing the relevance of SSRIs in the care of many pediatric psychiatric disorders. As evidenced-based care evolves over the coming decade, advances in the neurobiology, genetics, processing, and mechanisms of action by serotonin and its neurotransmission. The progress in the development and assessment of screening and diagnostic measures is easily foreseen. Recently published harbingers document this process with or without NIMH funding.^79^

The challenges in the clinical use and research of SSRIs will be to parcel out the myriad factors influencing their implantation, addressed in this and a subsequent narrative review. The following table provides scientific empirical responses to considerations surrounding the use of SSRIs.

## Conclusion

The enigma of understanding human suffering has been a matter of debate dating to the inception of civilization, with different philosophical traditions and fraternities providing their descriptive interpretations, discourse, and interventions. These approaches, to a certain extent, allayed some of the problems and on many occasions did not. With the rise of continental philosophy and criticisms about limits to reasoning, Francis Bacon, known as the Father of Empiricism, admonished the philosophies that emphasized the importance of deductive reasoning. Instead, Bacon suggested that hypotheses must be tested through inductive means via observations, measurements, and experiments.

These methods have merit in studying unitary constructs; however, quantifying subjective illness with polygenic inheritance, pleiotropy, and heterotypic continuity is a daunting task complicated by many biases and fallacies that inspire contentions and debates. The significant heterogeneity in child and adolescent mental health disorders represents one of many confounders. The heterogeneity includes but is not limited to developmental aspects, heterotrophic continuity, and the complexities of understanding receptor’s ontogeny and myths associated with theoretical foundations. The accompanying misinterpretations of two decades of extensive studies have contributed to widespread confusion.

Recent articles targeting the monoamine hypothesis have further confounded and obfuscated these discussions. Consequently, many providers, clinicians, researchers, and the public remain perplexed about how to address the fundamental issues related to these widely prescribed medications. Therefore, compared to adult studies, trials involving adolescents and children related to SSRTs have yielded more significant data. When this data is explored and critiqued using advanced statistical analysis, it can provide a more robust understanding, potentially leading to better implementation in clinical settings. Although there are many nuances in receptor ontogeny, epigenetics, variabilities in alleles, etc., translating and applying this data to clinical phenotypes with multiple interacting variables and co-occurring disorders remain key and central to positive outcomes.

Therefore, clinicians must stay informed about the limits of empirical findings and utilize their ability to critically appraise the literature to best address the needs of patients and families. An open, nonjudgmental sequential stepped strategy, awareness of confounders, and maintaining a shared decision-making paradigm could provide better outcomes. Many critics argue that the term illness provides a better subjective understanding of human experiences as compared to disorder or disease without the risk of being too reductionistic. The openness to exploring alternative options is essential to practice when treatment resistance and non-response are encountered more often than expected. The debate presented above remains an ongoing process and is paramount to scientific discoveries using the best available evidence. However, a flexible treatment strategy for sufficient duration and dosage, within the clinician’s attributes and skills, remains central to reducing the mounting burden of mental health conditions.

In the second part of this narrative review, we shall develop this further to address nosology, developmental correlates, and comorbidity assessments, and delve deeper in screening for phenotypes that have robust outcomes for SSRIs. Testimony to SSRI medications’ benefits outweighing the risks is the fact that millions have decided to continue their use amidst all these debates. Individualizing treatment to address the multiple co-occurring interacting variables, with ongoing monitoring to adjust and modify, attests to the role of sustaining trusting, professional relationships with the clinician.
